# State Anxiety Is Associated with Cardiovascular Reactivity in Young, Healthy African Americans

**DOI:** 10.1155/2012/268013

**Published:** 2012-01-03

**Authors:** Mildred A. Pointer, Sadiqa Yancey, Ranim Abou-Chacra, Patricia Petrusi, Sandra J. Waters, Marilyn K. McClelland

**Affiliations:** ^1^Julius L. Chambers Biomedical/Biotechnology Research Institute, North Carolina Central University, Durham, NC 27707, USA; ^2^School of Business, North Carolina Central University, Durham, NC 27707, USA; ^3^Department of Psychology, North Carolina Central University, Durham, NC 27707, USA

## Abstract

Although several studies have shown that enhanced cardiovascular reactivity can predict hypertension development in African Americans, these findings have not been consistent among all studies examining reactivity and hypertension susceptibility. This inconsistency may be explained by the influence of anxiety (state and trait) on the blood pressure response to stress. Therefore, this study sought to determine whether anxiety is associated with blood pressure response to cold pressor (CP) and anger recall (AR) stress tests in young healthy African Americans. Modeling using state and trait anxiety revealed that state anxiety predicts systolic (SBP) and diastolic blood pressure DBP response to CP and AR (*P* ≤ 0.02). Interestingly, state anxiety predicted heart rate changes only to CP (*P* < 0.01; *P* = 0.3 for AR). Although trait anxiety was associated with SBP response to AR and not CP, it was not a significant predictor of reactivity in our models. We conclude that anxiety levels may contribute to the variable blood pressure response to acute stressors and, therefore, should be assessed when performing cardiovascular reactivity measures.

## 1. Introduction

Although enhanced cardiovascular reactivity is generally associated with future development of hypertension and other cardiovascular events [1–5], there are studies that have failed to show any relationship between reactivity to stress and future elevation of blood pressure [1, 6–12]. The reason for these inconsistent findings is unclear. Emerging evidence suggest that some stress tests may be better predictors of future cardiovascular events than other stressors [4, 5, 10]. For example, blood pressure response to arithmetic and star tracing stress tests predicted high blood pressure while reactivity to cold pressor stress test did not [13, 14]. Furthermore, metanalysis of studies that assessed mental stress tests and hypertension development revealed variable success of mental tests in predicting hypertension. Among the different types of mental stressors, cognitive mental stressors were more consistent in predicting hypertension compared to emotion evoking, interview, and public speaking stressors [10]. The inconsistencies in prediction do not appear to be explained by differences in the type (mental, physical, or psychophysical) of stress tests for there is inconsistent predictability even among the types of stressors.

Another possible explanation for the inconsistencies in reactivity prediction of adverse cardiovascular outcomes is the interaction of psychosocial factors with cardiovascular responses to acute laboratory stressors. Anxiety is one such psychosocial factor that may determine reactivity responses. Metanalysis of over 700 studies revealed that chronic (trait) anxiety is associated with decreased cardiovascular reactivity [15]. In contrast, a study of young European population revealed that acute (state) anxiety was associated with significantly increased reactivity to cold pressor test but not mental stress test [16]. These observations, when taken together, suggest that individuals may be inaccurately identified as hyperresponsive if anxiety is not considered as a confounder in the reactivity response to acute laboratory stress tests. Consequently, inaccurate assessment of increased reactivity due to the interaction of anxiety with acute stressors may explain the inconsistent reports of increased risk of hypertension with increased reactivity.

This study sought to investigate whether (1) anxiety determined the blood pressure response to stress tests and (2) anxiety differentially influenced blood pressure response to anger recall and cold pressor stress tests in African Americans. We chose to study African Americans for several reasons: (1) this group is characterized as hyperresponsive to stress [7, 17–20], (2) several reports have failed to find increased reactivity in this population [4, 8, 21–23], and (3) psychosocial factors, including anxiety, are significantly associated with blood pressure in this population [24–29]. We report that state (in the moment) anxiety was significantly associated with blood pressure response to both stressors (anger recall and cold pressor stress tests) in this population. These results support the idea that identification of hyperresponders to acute stress tests among African Americans must take into account anxiety levels before determining whether an individual has increased reactivity to acute stress and/or that anxiety may play an important role along with reactivity response in hypertension development. However, our results do not support the idea that anxiety differentially impacts reactivity response to psychological, psychophysical, and physical stressors.

## 2. Methods

### 2.1. Participants and Procedures

A sample of 179 (116 males, 63 females) participants of African descent were recruited to the study. All study procedures and materials were approved by and in compliance with the North Carolina Central University institutional review board. Eligibility criteria for entry were (1) be 18 to 65 years old (2) being a student or employee at North Carolina Central University or living in the surrounding regions of Durham, Orange and Wake counties, (3) having no diagnosed cardiovascular disease (self-reported), and (4) not taking any hypertensive medication. These regions of Durham, Orange, and Wake counties make up the North Carolina Triangle region that is in the stroke belt (e.g., a geographic region with a higher occurrence of stroke) [30]. Of these 179, only 50 are reported in the current report; these were selected based on the type of mental stress used. The 50 participants reported here met the following criteria: (1) completed both the trait anxiety scale and the state anxiety scale, and (2) were between the ages of 18 and 40 years old. Study participants were scheduled at either 9 am or 1 pm for the three-hour study protocol. After receiving informed consent, trained staff measured blood pressure by sphygmomanometer method with a GE Dinamap Pro 100 automatic model and a cuff size appropriate for the body size. Each participant was allowed five minutes to sit quietly before taking the first resting parameters. The Dinamap was set to assess systolic blood pressure (SBP) and diastolic blood pressure (DBP) at one-minute intervals for the resting measurements as well as during the two acute stressor tasks. State anxiety survey was administered prior to baseline blood pressure measurements. Following resting blood pressure and heart rate measurements, participants were administered the cold pressor test, consisting of submersion of the hand in ice cold water for three minutes followed by a five-minute recovery period. A psychological stressor, anger recall, was given only after blood pressures and heart rate returned to baseline resting values. Anger recall stress consisted of 5 minutes of contemplating an event that evoked anger, 5-minute discussion about the event, and 5-minute recovery period. Trait anxiety survey was administered following the completion of the anger recall stressor. Cardiovascular reactivity was calculated as the difference between the average baseline prestressor blood pressure and the average change in blood pressure over the 5-minute stress period.

The study protocol consisted of state anxiety assessment, resting BP measurement, second resting BP measurement, cold pressor stressor, third resting BP measurement, anger recall stressor, trait anxiety assessment, recording medical history, body mass index measurement, and completing a demographics questionnaire.

### 2.2. Psychosocial Anxiety Measure

Anxiety was assessed using the state-trait anxiety inventory [31]. State anxiety is defined as an acute response to a threatening or challenging situation, while trait anxiety is defined as a stable and enduring tendency to be anxious. Each subscale is a 20-item self-report inventory. Each item is rated on a four-point scale (1 = almost never, 4 = almost always). Items from each subscale are summed to create a total state anxiety score and a total Trait Anxiety score. Higher scores on the state anxiety subscale indicate greater anxiety at the present time; higher scores on the trait anxiety subscale indicate greater anxiety, in general. The state anxiety subscale has an alpha coefficient of  .87, and the trait anxiety has an alpha coefficient of  .88, indicating good (since  .80 or greater) internal consistency in this sample.

## 3. Statistics

Data analysis was performed using SAS 9.1.3 for Windows [32]. Scoring of the psychosocial scales Spielberger State Trait Anxiety Inventory utilized scoring protocols documented in prior research as indicated above and were confirmed with factor analysis. Cronbach's alpha values were confirmed as reported above. Mean, standard deviation, standard error, median, and quartile calculations provide data reductions for SBP, DBP, and other clinical measures with multiple measurements. Regression models, goodness of fit, multivariate parameter estimates, and confidence intervals were evaluated for each stressors impact on SBP, and DBP. Two participants did not complete the cold pressor stressor; thus, the sample size is 48 for the cold pressor cardiovascular reactivity and 50 for the anger recall cardiovascular reactivity.

## 4. Results

Table 1 shows the baseline characteristics of the study sample. The African American study samples are relatively young (median age of twenty-one years) with normal BMI (median BMI was 26.2 kg/m^2^; normal BMI is 25–30 kg/m^2^) and waist circumference normal values of less than 102 for males and 88 cm for females [33–35]. This group also had normal cholesterol (less than 200 mg/dL), triglycerides (less than 150 mg/dL), glucose (less than 126 mg/dL), and insulin (<10 mIU) levels. This group was normotensive with median systolic (SBP) and diastolic (DBP) blood pressures of 114 and 70 mmHg, respectively.

A summary of the cardiovascular reactivity responses to AR and CP is shown in Table 2. Cardiovascular reactivity was defined as the change in cardiovascular parameters (SBP, DBP, mean arterial pressure (MAP), and heart rate (HR)) following the induction of a stress stimulus compared to baseline cardiovascular parameters. Both the CP stressor and the AR stressor produced significant rises in SBP, DBP, and HR. All of the values returned to baseline during the recovery period except for SBP during the CP recovery period. Repeated measures ANOVA and the multiple comparison test, Student-Newman-Keuls test, verified that the two stressor tasks (CP and AR) produced statistically significant increases (*P* < 0.0001) in the cardiovascular parameters in comparison to the resting measurements. The results suggest that both tasks induced stress-related cardiovascular activity.

 We examined Pearson's correlations of state anxiety, trait anxiety, age, BMI, and resting cardiovascular measures with the cardiovascular reactivity parameters. Trait anxiety had a statistically significant, positive correlation with state anxiety (*n* = 50; Pearson's *r* = 0.47; *P* < 0.001, two-tailed Pearson's correlation). State anxiety had statistically significant, positive correlations with CP reactivity response for SBP (average change; *n* = 48; Pearson's *r* = 0.37; *P* = 0.01), DBP (average change; *n* = 48; Pearson's *r* = 0.40; *P* = 0.005). Similarly, state anxiety was highly correlated with the AR reactivity response for SBP (*n* = 50; Pearson's *r* = 0.34; *P* = 0.015) and DBP (*n* = 50; Pearson's *r* = 0.35; *P* = 0.013). State anxiety exhibited differential effects on HR response to CP and AR. Specifically, state anxiety was significantly associated with HR change to CP (average change; *n* = 48; Pearson's *r* = 0.37; *P* = 0.009) but was not related to HR changes to AR (*n* = 50; Pearson's *r* = 0.14; *P* = 0.30).

Trait anxiety also had a differential association with reactivity to CP versus AR. Trait anxiety had a nonsignificant correlation with SBP (*n* = 48; Pearson's *r* = 0.23; *P* = 0.12) but a positive, significant correlation for DBP (*n* = 48; Pearson's *r* = 0.34; *P* = 0.02) reactivity response to CP. As for AR, trait anxiety was positively and significantly correlated with SBP (*n* = 50; Pearson's *r* = 0.35; *P* = 0.012) but was not significantly correlated with DBP (*n* = 50; Pearson's *r* = 0.23; *P* = 0.11).

Age, BMI, and resting cardiovascular measures had nonsignificant correlations with both CP reactivity and AR reactivity as measured by SBP, DBP, and HR changes. Resting SBP was only associated with MAP changes with CP and AR stress tests (Pearson's *r* = 0.33; *P* < 0.02 and *r* = 0.38; *P* < 0.01, resp.).

 The following variables were evaluated in the stepwise procedure: state anxiety, trait anxiety, resting cardiovascular measures, body mass index, and age. The variable selection results of the stepwise algorithm suggested the use of state anxiety for a parsimonious model of both cold pressor cardiovascular reactivity as well as anger recall cardiovascular reactivity.

Our next step was to evaluate regression models to predict cardiovascular reactivity with state anxiety as the independent variable. The model for predicting the CP increase in SBP (see Table 3) had an *r* ^2^ of 0.14 and state anxiety as a significant parameter (*P* = 0.009). State anxiety was also a significant independent variable (*P* = 0.005) in the model of the CP change in DBP (*r* ^2^ = 0.16). Similar results were found with the model for predicting the change in cardiovascular reactivity for the AR stressor as shown in Table 4. State anxiety was a significant parameter for the change in SBP (*P* = 0.015, *r* ^2^ = 0.12) and DBP (*P* = 0.013, *r* ^2^ = 0.12).

Each model was scrutinized to verify adherence to the assumptions of regression modeling (linear relationship between independent variables and dependent variables, homoscedasticity of the errors, and errors are independent and normally distributed). Plots of residuals of each model against the predicted values were checked. Shapiro-Wilke statistics did not reject the null hypothesis of normal distribution of the residuals.

## 5. Discussion

This study investigated whether anxiety differentially affects cardiovascular reactivity to cold pressor and anger recall stress tests in a sample of young, healthy, community dwelling African American adults, a population prone to develop cardiovascular disease. Importantly, we show that the state (at the moment) anxiety was significantly associated SBP and DBP responses to both cold pressor and anger recall laboratory stress tests in this population. In contrast to blood pressure, state anxiety differentially predicted HR response to CP but not AR. On the other hand, chronic (trait) anxiety was not a significant predictor of reactivity in our statistical models. We interpret these results to mean that the state of anxiety at the time of the stressor must be considered when assessing cardiovascular reactivity to laboratory stress tests. Failure to consider state anxiety as a confounder of reactivity responses may lead to misidentifying some individuals as hyperresponders when compared to others. Misidentification of individuals may contribute in part to the inconsistent findings of increased reactivity in African Americans and to the inconsistent prediction of hypertension in those with increased vascular reactivity. Alternatively, these results can be interpreted to mean that the interaction of state anxiety and cardiovascular reactivity may be important determinants of hypertension development in African Americans.

Anxiety, chronic anxiety in particular, has been linked to the development of disease [36]. Contrary to what would be expected, chronic anxiety has been shown to be negatively associated with cardiovascular reactivity [15]. Although chronic anxiety and cardiovascular reactivity associations have been studied, few studies have investigated the role of state anxiety in determining blood pressure response to stress. State anxiety is important in predicting the DBP “white coat” response and is effective in predicting ambulatory evening systolic blood pressure in young black males [24]. Studies that have investigated the effect of state anxiety on reactivity did not include African Americans [16, 37]. Our study provides evidence that young healthy African Americans who are anxious prior to the stress tests are likely to have higher blood pressure responses to the stress. Thus, variability in hyperresponsiveness response to laboratory stress tests in African Americans may be due in part to a failure to consider state anxiety as a confounder.

Although many studies have shown that enhanced cardiovascular reactivity predicts hypertension development and other cardiovascular events [1–5], there are several studies that failed to show any relationship between reactivity to stress and hypertension development [1, 6–12]. The reason for the variable predictive response to laboratory stress tests is unclear. The inconsistency may be a consequence of the type of stressors used [4, 5, 38, 39] and the interaction of psychosocial factors with blood pressure response to the stressor [40–42]. Psychological stress tests may be better than cold pressor stress tests at predicting future cardiovascular events [4, 5, 22, 43, 44]. This differential effect of stressor type and hypertension development may be a consequence of differential psychosocial factor interaction with stressors. In a study of a European population, psychosocial factors appear to have a greater impact on reactivity to cold pressor than reactivity to mental stress [16]. Our study compares the impact of anxiety on reactivity to cold pressor and anger recall in African Americans. Anxiety was significantly associated with blood pressure response to both stressors in this population.

Another explanation for the inconsistent findings of increased risk of hypertension development with increased reactivity is the interaction of psychosocial factors with cardiovascular reactivity to promote hypertension development. For example, studies show that hostility, depression, and anger contribute to increased reactivity [40, 45, 46]. Psychosocial factors also are associated with increased incidence of cardiovascular events [47–50] as well as the development of cardiovascular disease [51, 52]. We show that the psychosocial factor, anxiety, can influence the reactivity response to acute stress. However, because the study design was cross-sectional, it could not be determined whether state anxiety interaction with cardiovascular response to acute stress predicts hypertension development.

This study shows that the current state of anxiety was significantly associated with blood pressure response to laboratory stress tests. Consequently, anxiety levels should be assessed when using acute laboratory stress tests for identification of those with increased cardiovascular reactivity. Further studies are needed to determine whether reactivity normalized to anxiety increases the accuracy in identifying hyperresponders and, subsequently, predicting future hypertension development.

## 6. Limitations

The small sample size is a major limitation of this study. These findings need to be validated in a larger cross sectional population of African Americans. Additionally, our study design did not allow us to determine whether the impact of anxiety on blood pressure response to acute stressor is unique to anxiety or whether other psychosocial factors similarly influence blood pressure response to acute stress in our cohort. Another limitation of the study is that adrenergic system activation was not measured; consequently, it could not be determined if the two stressors differentially activated the beta or alpha-adrenergic receptor pathways. This information would be helpful in future longitudinal studies that will address how activation of the alpha and beta adrenergic receptors pathways ultimately leads to hypertension development and the attending cardiovascular disease. A longitudinal study design will also help to address the question of whether inclusion of anxiety enhances the ability of increased reactivity to predict future elevations of blood pressure.

## Figures and Tables

**Table 1:** Participants traditional cardiovascular risk factors.

	Total *N*	Mean (SD)
		Median (Q1, Q3)
Age	50	23.6 (6.7)
		21 (19, 25)
SBP	50	114.3 (11.9)
		112.6 (105.9, 123.6)
DBP	50	69.9 (7.4)
		68.9 (64.3, 72.7)
MAP	50	87.2 (7.2)
		86.0 (63.0, 75.8)
HR	50	70.3 (9.8)
		69.4 (63.0, 75.8)
HOMA	21	2.1 (2.0)
		2.0 (0, 2.0)
Glucose	30	83.4 (14.5)
		83.5 (79.0, 92.0)
Insulin	21	10.9 (8.5)
		9.2 (5.1, 14.4)
BMI	50	28.1 (7.1)
		26.2 (23.0, 33.2)
Waist	40	81.1 (16.2)
		77.6 (71.4, 89.0)
Cholesterol	30	169.1 (36.1)
		170.5 (146, 195)
HDL	30	54.5 (15.3)
		53.5 (42, 65)
LDL	30	99.3 (36.0)
		97.5 (69, 123)
Triglycerides	30	76.5 (30.5)
		65.5 (54, 94)

**Table 2:** Cardiovascular reactivity responses to cold pressor and anger recall.

	Cold pressor^a^	Anger recall^a^
	Mean (SD)	Mean (SD)
	Median(Q1, Q3)	Median(Q1, Q3)
	*N*	*N*
ΔSBP	17.0 (9.9)	9.9 (8.8)
	16.3 (11.3, 23.5)	10.1 (3.4, 14.6)
	48	50
ΔDBP	12.5 (7.8)	6.4 (5.4)
	12.0 (7.1, 18.3)	5.8 (2.9, 10.6)
	48	50
ΔMAP	12.2 (7.7)	6.5 (5.2)
	11.1 (6.2, 17.4)	5.8 (2.9, 9.9)
	48	50
ΔHR	6.7 (7.1)	4.7 (5.8)
	6.5 (2.1, 9.9)	4.7 (0.8, 8.2)
	48	50

^a^All values were significantly different from baseline (*P* < 0.0001).

**Table 3:** Parsimonious linear regression models for cardiovascular reactivity to cold pressor test.

	*R* ^2^	*df*	*F*	*P*	Variables	*β*	*t*	*P*
ΔSBP	0.14	1,46	7.35	0.009	State anxiety	8.72	2.71	0.009
ΔDBP	0.16	1,46	8.63	0.005	State anxiety	7.36	2.94	0.005
ΔMAP	0.15	1,46	7.94	0.007	State anxiety	7.00	2.82	0.007
ΔHR	0.14	1,46	7.48	0.009	State anxiety	6.28	2.73	0.009

The models explained 14% of the variance for SBP, 16% for DBP, 15% for MAP, and 14% for HR.

**Table 4:** Parsimonious linear regression models for cardiovascular reactivity to anger recall.

	*R* ^2^	*df*	*F*	*P*	Variables	*β*	*t*	*P*
ΔSBP	0.12	1,49	6.39	0.015	State anxiety	7.33	2.53	0.015
ΔDBP	0.12	1,49	6.69	0.013	State anxiety	4.59	2.59	0.013
ΔMAP	0.20	1,49	11.67	0.001	State anxiety	5.60	3.42	0.001
ΔHR	0.02	1,49	1.08	0.303	State anxiety	2.02	1.04	0.303

The models explained 12% of the variance for SBP, 12% for DBP, 20% for MAP, and 2% for HR.
